# Elastography for the evaluation of thyroid nodules in pediatric patients

**DOI:** 10.1590/0100-3984.2018.0034

**Published:** 2019

**Authors:** Gustavo Bittar Cunha, Luciana Cristante Izar Marino, André Yamaya, Cristiane Kochi, Osmar Monte, Carlos Alberto Longui, Adriano Namo Cury, Eduardo de Faria Castro Fleury

**Affiliations:** 1 Irmandade da Santa Casa de Misericórdia de São Paulo, São Paulo, SP, Brazil.

**Keywords:** Thyroid neoplasms, Child, Thyroid nodule/diagnostic imaging, Elasticity imaging techniques, Ultrasonography, Neoplasias da tireoide, Criança, Nódulo da tireoide/diagnóstico por imagem, Técnicas de imagem por elasticidade, Ultrassonografia

## Abstract

**Objective:**

To evaluate the usefulness of elastography (using manual compression) as an additional diagnostic tool for children and adolescents with thyroid nodules.

**Materials and Methods:**

This was a prospective study conducted between September 2012 and August 2013 at a hospital in Brazil. We performed elastography, ultrasound, and fine-needle aspiration biopsy in 32 patients between 6 and 18 years of age who had, in total, 38 thyroid nodules.

**Results:**

The elastography findings correlated with the histopathological diagnosis in 78.5% of cases. In three patients, an unnecessary thyroidectomy could have been avoided if the elastography results had been prioritized. Only one malignant thyroid nodule was found to show high elasticity.

**Conclusion:**

Our results suggest that high elasticity of a nodule on elastography is associated with a low risk of thyroid cancer. If further confirmed in other studies, elastography may prove useful as a complementary test for screening thyroid nodules in children.

## Abstract (pt)

**Objetivo:**

Avaliar a utilidade da elastografia (usando compressão manual) como uma ferramenta adicional de diagnóstico em crianças e adolescentes com nódulos da tireoide.

**Materiais e Métodos:**

Este foi um estudo prospectivo realizado entre setembro de 2012 e agosto de 2013 em um hospital no Brasil. Realizamos elastografia, ultrassonografia e biópsia por aspiração com agulha fina em 32 pacientes com idade entre 6 e 18 anos que tinham, no total, 38 nódulos tireoidianos.

**Resultados:**

Os achados da elastografia em relação ao histopatológico foram corretos em 78,5% dos casos. A elastografia poderia ter evitado a tireoidectomia desnecessária de três pacientes. Apenas um nódulo maligno da tireoide foi classificado como E1.

**Conclusão:**

Nossos resultados sugeriram que a alta elasticidade de um nódulo na elastografia está associada a um baixo risco de câncer de tireoide. Caso se confirme em outros estudos, a elastografia pode ser útil como teste de triagem complementar de nódulos tireoidianos em crianças.

## INTRODUCTION

Thyroid nodules are rare in children, with an incidence of 1.0-1.5%, although they are seen in up to 13% of adolescents and young adults^(^^1^^,^^2^^)^. However, the risk of malignancy among children and adolescents with thyroid nodules is high, the incidence of thyroid carcinomas in pediatric patients being 18.0-26.7%^(^^3^^)^, with a risk of recurrence as high as 39%^(^^4^^)^. The incidence of thyroid cancer is 1:1,000,000 among children under 10 years of age, 1:200,000 among individuals 10-14 years of age and 1:75,000 among individuals 15-19 years of age. After puberty, girls are four times more likely to have thyroid cancer than are boys, whereas the female:male ratio is 1:1 in the prepubertal period^(^^5^^)^.

Children are often diagnosed with advanced thyroid cancer, 40-90% of cases presenting with lymph node involvement and 20-30% presenting with distant metastases^(^^6^^)^. A recent study of children with suspicious nodules showed rates of malignancy and metastasis of 25% and 44%, respectively^(^^7^^)^. Thorough investigation of suspicious nodules that might require surgery is recommended to ensure early identification of malignancy and to avoid unnecessary thyroidectomy in those with benign nodules^(^^8^^)^.

The protocol for the diagnosis of thyroid nodules includes physical examination, laboratory testing, fine-needle aspiration biopsy (FNAB), and thyroid ultrasound^(^^2^^)^. However, the most appropriate investigations of thyroid nodules are still debated. Ultrasound is considered more sensitive than physical examination for the detection of thyroid nodules, although the former has low specificity for differentiating between malignant and benign nodules^(^^9^^)^. The ultrasound criteria for malignant thyroid nodules include marked hypoechogenicity, indistinct or spiculated margins, microcalcifications, intranodular vascularity, and a taller-than-wide shape, although those criteria have a wide range of sensitivities (55-95%) and specificities (52-81%) for identifying and differentiating between malignant or benign^(^^10^^)^.

Although FNAB is thought to be the most accurate method for diagnosing thyroid nodules, it is particularly difficult to perform the procedure in children because of the discomfort associated with the biopsy. In addition, in 20-40% of cases, the cytological findings following FNAB are indeterminate or the specimen is insufficient for a definite diagnosis. An accurate diagnosis is often made only after histological evaluation^(^^11^^)^. The nodule size that warrants a biopsy is still debated. In adults, biopsy is recommended for nodules ≥ 1.0 cm, whereas it is advisable to perform biopsy on smaller nodules (those ≥ 0.5 cm) in children^(^^2^^)^, because of the high incidence of malignancy among children.

Elastography is a promising new technique for the ultrasound evaluation of thyroid nodules^(^^12^^,^^13^^)^. The technique relies on software that is available for conventional ultrasound devices, and it evaluates the different types of tissue, according to variations in their stiffness. It is based on the principle that benign lesions are usually more elastic than are malignant lesions, and elastography data can facilitate the differential diagnosis of clinical pathologies, such as thyroid nodules^(^^14^^)^.

Ultrasound elastography is a noninvasive, painless imaging tool, based on the estimation of the mechanical properties (elasticity) of the tissue, that provides additional and clinically relevant information^(^^15^^)^. One recent study showed that elastography can increase the accuracy of ultrasound in thyroid nodule differentiation and suggested that it is the best noninvasive procedure available, comparable to FNAB for the evaluation of thyroid nodules, regardless of the nodule size, provided that the nodule is solid and without calcification^(^^16^^)^.

Ultrasound elastography can be useful to distinguish between benign and malignant thyroid nodules^(^^16^^)^. Because malignant nodules exhibit stiffness on elastography, this approach is also believed to have diagnostic value in the identification of malignant lesions. In addition, refined elastography approaches can provide an objective ratio (or index) as a quantitative indicator to compare the stiffness of a lesion with that of normal tissue or with a direct power unit^(^^17^^)^.

Elastography acquires information on the movement of the tissue in response to the application of a small amount of pressure^(^^15^^)^. In softer tissues, the applied pressure causes the tissue to compress more, whereas harder tissues compress less^(^^15^^)^. This compressibility of the tissue (its stiffness) is known as strain and is presented as a color map called an "elastogram"^(^^15^^)^. Colors around and within the nodules are evaluated and visually scored according to the 4-5-scale scoring system, the regions of interest being specified as the target region and the adjacent reference region. A score of 4 indicates no elasticity in the target region (the nodule), and a score of 5 indicates no elasticity in the target region or in the adjacent reference region (the area showing posterior shadowing). The strain ratio is calculated automatically, and a higher strain ratio translates to a higher probability of malignancy^(^^18^^)^. Another form of evaluating the elastography data is by calculating the thyroid strain ratio to perform an objective, semiquantitative analysis of the tissue stiffness^(^^19^^)^.

Although many ultrasound elastography reports have referred to "cytologically indeterminate" thyroid nodules, it is practical to define the ultrasound indications for the application of elastography during ultrasound scanning of thyroid nodules^(^^20^^)^. Elastography can be easily used as an adjunctive noninvasive tool for B-mode ultrasound of indeterminate nodules, which are common^(^^20^^,^^21^^)^. However, it is not yet known whether this approach is suitable for the risk stratification of such nodules^(^^20^^,^^21^^)^.

In general, there are two ultrasound methods used for determining thyroid nodule stiffness^(^^22^^)^: strain elastography, in which force is applied to the tissue by manual compression and the tissue deformation is parallel to the direction of the force; and shear-wave elastography (SWE), in which a push beam is created and the tissue deformation is perpendicular to the direction of the force.

In children and adolescents with thyroid nodules, differentiating between benign and malignant nodules is vitally important. We evaluated the usefulness of strain elastography as an additional diagnostic tool for children who present with thyroid nodules.

## MATERIALS AND METHODS

This was a prospective study conducted between September 2012 and August 2013 at a hospital in Brazil. The research ethics committee of the institution approved the study (Authorization no. 01687412200005479). The parents or legal guardian of each participant gave written informed consent, and all of the participants provided assent.

We evaluated 35 patients with thyroid nodules who had been treated in the pediatric endocrinology department of our hospital. In total, 42 thyroid nodules were identified on physical examination or cervical ultrasound. We excluded three patients with a total of four nodules: in one patient (with two nodules), the FNAB results were inconclusive; in another patient, the initial evaluation showed that the nodule had invaded the muscle but subsequent evaluations showed that it had regressed; and one patient withdrew from the study. Therefore, the final sample comprised 32 patients with a total of 38 thyroid nodules.

Laboratory evaluations of thyroid function (to determine circulating levels of thyroid-stimulating hormone, free thyroxine, thyroid peroxidase antibody, antithyroglobulin antibody, and calcitonin) were conducted. Patients with euthyroidism were referred for ultrasound, elastography, and FNAB. All examinations were conducted by the same radiologist who, at the time of the study, had nine years of experience in thyroid imaging. The specimen obtained by FNAB was evaluated by a cytologist with more than 30 years of experience in cytological analysis.

The Bethesda System for Reporting Thyroid Cytopathology was used in order to categorize the biopsy findings as nondiagnostic (category I), benign (category II), atypia of undetermined significance (category III), suspicious for follicular neoplasm (category IV), suspicious for malignancy (category V), or malignant (category VI). Thyroidectomy was recommended for patients in whom the cytology was potentially non-benign (thyroid nodules classified as Bethesda category IV, V, or VI).

Ultrasound and elastography were performed on an ultrasound system with a 5-14 MHz multifrequency transducer (Sonix SP; Ultrasonix Medical Corporation, Vancouver, Canada). For the elastography study, we used special software developed for the Ultrasonix system (version 3.0.2 [beta 1]; Ultrasonix Medical Corporation). The radiologist in charge of the study obtained the rights to use the software for experimental research during the study period. Elastography was performed during the ultrasound examination, with the same real-time instrument and the same probe. The operator highlighted a box (region of interest) that included the nodule to be evaluated.

**Figure 1:**
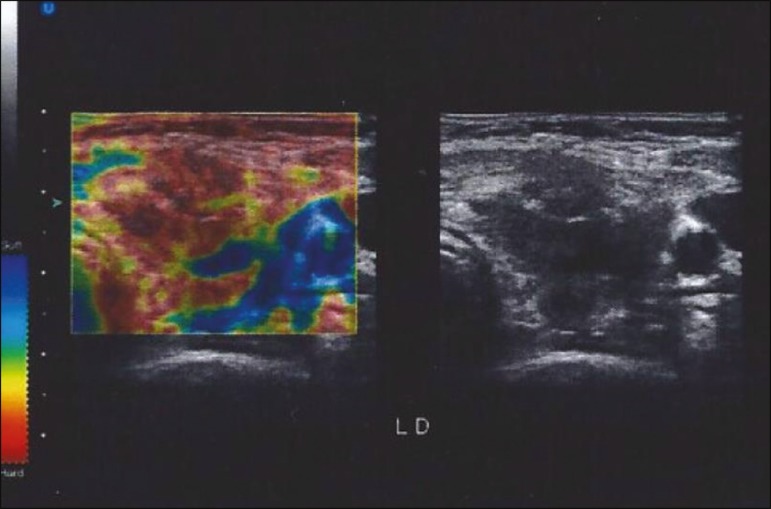
Green light appears to the left of the ultrasound image when the degree of manual compression is correct.

The patient was placed in the supine position with the neck in hyperextension, and the ultrasound transducer was held perpendicular to the region of interest. For elastography, slight, continuous manual compression was exerted with the transducer on the thyroid nodules until resistance could be felt. When resistance was sensed, the operator relaxed the hand holding the transducer so as to provide spontaneous decompression of the thyroid nodule. It is important that the pressure level remains constant throughout the examination. The operator knows that the degree of manual compression is correct when a green light appears to the left of the ultrasound image, as shown in Figure 1. The possibility to select the region of interest for the elastography analysis allowed accurate screening, even of small nodules, regardless of the position of the nodule within the thyroid gland.

The principle of elastography is to acquire two ultrasound images (one before tissue compression and one after) and track tissue displacement by assessing the propagation of the imaging beam. The use of dedicated software allows tissue distortion to be measured accurately. This technique is easy to perform. In the present study, examination times ranged from 30 s to 2 min. All examinations were performed by the same operator, who was blinded to the cytology results. The elastography software provides elasticity information in various colors. In descending order of elasticity, the colors are blue, green, yellow, and red.

**Figure 2:**
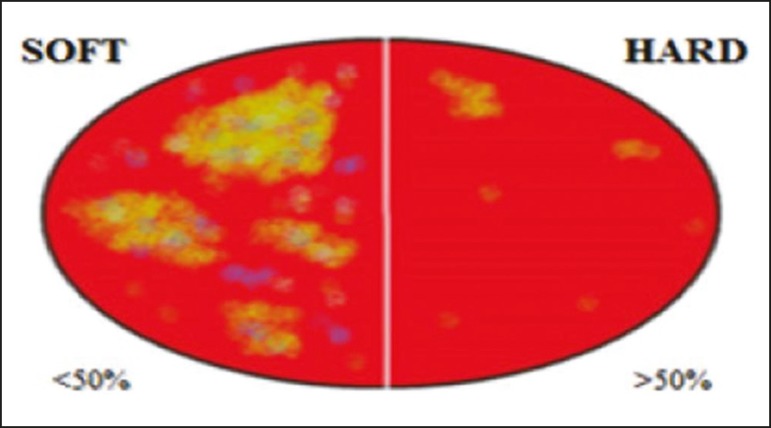
Classification of thyroid nodules by elastography. A classification of E1 indicated an elastic (probably benign) lesion, and a classification of E2 indicated a hard (probably malignant) lesion.

**Figure 3:**
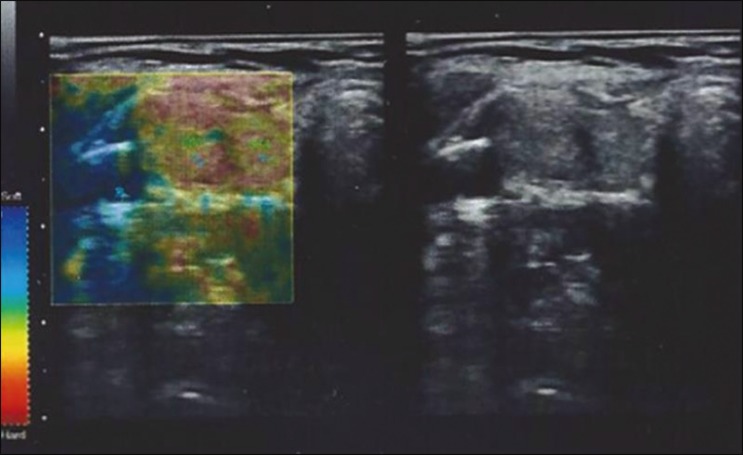
Elastography image of a thyroid nodule classified as E1.

**Figure 4:**
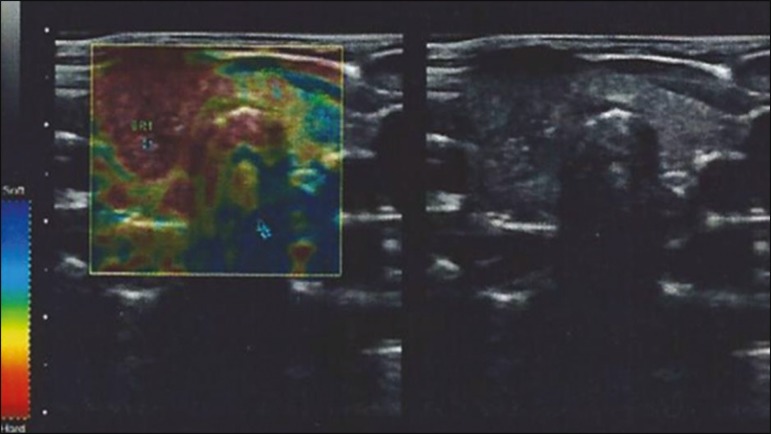
Elastography image of a thyroid nodule classified as E2.

In this study, elastography data were evaluated with a scoring system, modified from the Azizi et al.^(^^23^^)^ classification, in which each thyroid nodule was given a score of E1 or E2. As illustrated in Figures 2, 3, and 4, if < 50% of a nodule appeared in red, it was classified as E1 (elastic), whereas a nodule in which > 50% of the area appeared in red was classified as E2 (stiff).

The Bethesda cytology classification was used, and thyroidectomy was recommended for patients with thyroid nodules classified as Bethesda IV, V, or VI. McNemar's test was used in order to determine the level of agreement between the elastography findings (benign or malignant) and the histological diagnosis.

## RESULTS

### Overview

We enrolled 32 patients with one or more nodules, referred for suspicious nodules detected by palpation or as incidental radiographic findings. The mean age of the patients was 12.9 years (range, 6-18 years) and the female:male ratio was 2.5:1. The sample comprised 23 female patients and 9 male patients, with mean ages of 13.5 and 11.0 years, respectively.

The nodules ranged in size from 0.5 cm to 4.3 cm, and all were submitted to FNAB. Of the 32 patients, 12 had cytological abnormalities concerning for cancer and were referred for total thyroidectomy. Overall, seven patients were diagnosed with thyroid cancer, corresponding to an incidence of 22%. The histological diagnosis was papillary thyroid carcinoma in six of those patients and medullary thyroid carcinoma in one.

The patients with cytologically benign nodules agreed to long-term surveillance with annual cervical ultrasound examinations. If there were changes in the ultrasound features of a nodule, FNAB was again performed. At this writing, there have been no changes in the diagnosis of any of the benign nodules.

The mean age of the seven patients diagnosed with thyroid cancer was 12 years (range, 7-18 years). Six were female and one was male. In the sample as a whole, the test for antibodies was positive in 11 patients, only one of whom was among those diagnosed with thyroid cancer. The patient who was diagnosed with medullary thyroid carcinoma had elevated calcitonin levels: 3770 pg/mL (reference value: < 8.4 pg/mL).

### Thyroid nodules

On the basis of the FNAB results, 24 (63%) of the 38 thyroid nodules were classified as Bethesda II, 6 (16%) were classified as Bethesda IV, 7 (18%) were classified as Bethesda V, and 1 (3%) was classified as Bethesda VI. All thyroid nodules classified as Bethesda V and VI were found to be malignant in the postoperative pathology. Of the six thyroid nodules classified as Bethesda IV, five were benign and one was malignant. Of the 38 thyroid nodules evaluated, 18 (47%) were located in the right lobe; 17 (45%) were located in the left lobe; 2 (5%) were located in the isthmus; and 1 (3%) was diffuse. The two nodules in the isthmus were both benign, whereas the nodule with a diffuse appearance was malignant. Four of the nodules in the right lobe were malignant, as were four of those in the left lobe. All of the thyroid nodules (whether malignant or benign) ranged in size from 1.3 cm to 1.6 cm.

### Elastography features

Of the 14 thyroid nodules evaluated by histology, 5 (35.7%) were benign and 9 (64.3%) were malignant. McNemar's test showed that the elastography findings correlated with the histopathological diagnosis in 78.5% of the cases-in 21.4% of the benign nodules and 57.1% of the malignant nodules. Only one malignant nodule (the one diagnosed as medullary thyroid carcinoma) was classified as E1. In that particular case, the patient had a high level of calcitonin, as well as another thyroid nodule, which was classified as Bethesda V. Of the five thyroid nodules characterized as benign by histology, three were classified as E1.

As in studies involving much larger samples (of adults), the fact that the vast majority of patients with benign cytology do not undergo surgery precludes an absolute assessment of the accuracy of biopsy. However, of the 24 thyroid nodules characterized as benign after FNAB, the elastography result was classified as E1 in 22 (92%). Among the six patients referred for surgery after FNAB findings suspicious for follicular neoplasm (Bethesda IV), the elastography result was classified as E1 in three, and the histology confirmed the thyroid nodules to be benign in all three cases.

## DISCUSSION

Although this study was carried out between September 2012 and August 2013, it is still of great importance because there is a lack of articles evaluating the use of elastography in thyroid nodules with a focus on children and adolescents. Using elastography in real time, we observed that thyroid nodules with greater elasticity are at a lower risk of malignancy, as evidenced by the fact that three of the five nodules with benign histology were classified as E1. In addition, elastography classified only one malignant nodule as E1. However, that patient had a final diagnosis of medullary thyroid carcinoma, and it is known that up to 50% of such nodules exhibit greater elasticity^(^^24^^)^.

Of the six patients with FNAB findings suspicious for follicular neoplasm (Bethesda IV thyroid nodules), three had elastography results classified as E1 and a histological diagnosis of benign nodules. Therefore, those three patients were referred for thyroidectomy unnecessarily.

Although there have been a number of scientific reports about the diagnostic value of elastography in adults, only one study has addressed its use in children with thyroid nodules. In one recent study, Borysewicz-Sanczyk et al.^(^^25^^)^ employed elastography in their analysis of a total of 62 thyroid nodules in 47 children and adolescents: 37 females (age range, 9-18 years) and 10 males (age range, 7-17 years). The authors found elastography to have a high negative predictive value for a benign diagnosis. The first study to assess the role of elastography in the analysis of thyroid nodules was conducted in 2007 by Rago et al.^(^^16^^)^. The authors reported a positive predictive value of 100% and a negative predictive value of 98%. They concluded that elastography is a useful adjunctive tool for the diagnosis of thyroid cancer.

If larger studies confirm our results, elastography might allow children with elastic thyroid nodules to be placed in follow-up without invasive procedures or unnecessary thyroidectomies. However, the accuracy of elastography is limited by intra- and inter-operator variability^(^^26^^,^^27^^)^, which could be minimized by using SWE techniques^(^^28^^)^. Unlike strain elastography, SWE is a quantitative technique that is not operator-dependent, which avoids the limitations of strain elastography in evaluations of the head and neck, including the thyroid. Therefore, it is expected that SWE can provide more information^(^^28^^)^. However, to date, there have been few studies aimed at standardizing specific values for the various SWE techniques used in the evaluation of thyroid tissue^(^^24^^)^.

In a recent study, Duan et al.^(^^28^^)^ used two-dimensional SWE ultrasound in the evaluation of 137 nodules smaller than 10 mm, showing that SWE values were significantly higher in malignant lesions than in benign lesions. The SWE technique proved to be better than conventional ultrasound in differentiating between malignant and benign lesions. The combination of the two techniques had a specificity of 94.5%, sensitivity of 95.7%, positive predictive value of 89.8%, and negative predictive value of 97.7%.

Fukuhara et al.^(^^29^^)^, evaluating thyroid nodules with SWE, showed that the "shear-wave velocity" was significantly higher in papillary thyroid cancer than in normal tissues and benign tumors, and there was no significant difference between normal thyroid tissue and benign nodules. The authors also found that the results were most consistent for nodules larger than 20 mm.

In a large prospective study that evaluated 4550 nodules, of which 1305 were evaluated with strain elastography, the method showed a sensitivity of 74.2%, specificity of 91.1%, negative predictive value of 98%, positive predictive value of 37.4%, and accuracy of 90% to differentiate between malignant and benign nodules. In the present study, we concluded that hard nodules should always be considered suspicious for malignancy. However, nodules that are "less stiff" are also frequently diagnosed as carcinomas^(^^30^^)^.

## CONCLUSION

Given the conflicts related to the small number of studies addressing thyroid cancer in the pediatric population, we thought it important to improve the investigation of thyroid nodules in young patients. In the present study, the low number of false-negative elastography results implies that high elasticity of thyroid nodules is associated with benign histology.

Although markers such as microRNAs and somatic mutations of differentiated carcinomas have recently shown accuracy in the evaluation of thyroid nodules, ultrasound elastography could be incorporated into the routine evaluation of thyroid nodules, with greater ease and at a lower cost. However, further studies are needed in order to strengthen the role of elastography in the pediatric population, with a focus on FNAB cytology results classified as Bethesda III or IV, because those categories are responsible for the largest number of unnecessary thyroidectomies.
